# IFN-γ differentially modulates memory-related processes under basal and chronic stressor conditions

**DOI:** 10.3389/fncel.2014.00391

**Published:** 2014-11-20

**Authors:** Darcy Litteljohn, Eric Nelson, Shawn Hayley

**Affiliations:** ^1^Department of Neuroscience, Carleton UniversityOttawa, ON, Canada; ^2^Faculty of Medicine, University of TorontoToronto, ON, Canada

**Keywords:** depression, memory, hippocampus, cytokine, knockout mouse, monoamine, BDNF

## Abstract

Cytokines are inflammatory messengers that orchestrate the brain’s response to immunological challenges, as well as possibly even toxic and psychological insults. We previously reported that genetic ablation of the pro-inflammatory cytokine, interferon-gamma (IFN-γ), attenuated some of the corticosteroid, cytokine, and limbic dopaminergic variations induced by 6 weeks of exposure to an unpredictable psychologically relevant stressor. Presently, we sought to determine whether a lack of IFN-γ would likewise modify the impact of chronic stress on hippocampus-dependent memory function and related neurotransmitter and neurotrophin signaling systems. As predicted, chronic stress impaired spatial recognition memory (Y-maze task) in the wild-type animals. In contrast, though the IFN-γ knockouts (KOs) showed memory disturbances in the basal state, under conditions of chronic stress these mice actually exhibited facilitated memory performance. Paralleling these findings, while overall the KOs displayed altered noradrenergic and/or serotonergic activity in the hippocampus and locus coeruleus, norepinephrine utilization in both of these memory-related brain regions was selectively increased among the chronically stressed KOs. However, contrary to our expectations, neither IFN-γ deletion nor chronic stressor exposure significantly affected nucleus accumbens dopaminergic neurotransmission or hippocampal brain-derived neurotrophic factor protein expression. These findings add to a growing body of evidence implicating cytokines in the often differential regulation of neurobehavioral processes in health and disease. Whereas in the basal state IFN-γ appears to be involved in sustaining memory function and the activity of related brain monoamine systems, in the face of ongoing psychologically relevant stress the cytokine may, in fact, act to restrict potentially adaptive central noradrenergic and spatial memory responses.

## INTRODUCTION

It is widely accepted that the likelihood of developing depression, anxiety, and other psychological disorders is greatly influenced by exposure to stressors, particularly those of a chronic, unpredictable, and/or psychosocial nature (Hill et al., 2012). The prevailing view over many years has been that stressor-induced alterations of brain monoamine activity were largely responsible for the emotional and cognitive symptoms seen to predominate in these conditions (Schildkraut, 1965). While evidence continues to implicate monoaminergic neurotransmitter processes (Fava, 2003; Hamon and Blier, 2013), deficits in trophic growth factors such as brain-derived neurotrophic factor (BDNF), and even structural brain changes (e.g., impaired neurogenesis) have emerged as important players too in this regard (Pittenger and Duman, 2008; Calabrese et al., 2009; Mahar et al., 2014).

It’s become increasingly clear that cytokines and other elements of the inflammatory immune system contribute importantly to depression and other stress-related psychological disturbances (Miller et al., 2009; Anisman and Hayley, 2012). For instance, numerous studies have reported that pro-inflammatory cytokines, most notably interleukin-1-beta (IL-1β), IL-6, interferon-alpha (IFN-α), and tumor necrosis factor-alpha (TNF-α), are altered in major depression and stressor-based animal models (Dowlati et al., 2010; Liu et al., 2012; Dahl et al., 2014). Moreover, administration of these cytokines to rodents induced behavioral, hormonal, monoamine, and neuroplastic changes that are reminiscent of at least some depressive-like clinical changes (Myint et al., 2007; Anisman et al., 2008; Kaster et al., 2012; Sukoff Rizzo et al., 2012). The fact that anti-inflammatory and anti-cytokine treatments (e.g., minocycline, curcumin, cytokine-specific antagonists) were reported to lessen the neural and behavioral impact of stressor exposure further supports a link between cytokines and stressor pathology (Koo and Duman, 2008; Hinwood et al., 2012; Jiang et al., 2013; Krügel et al., 2013).

Interferon-gamma (IFN-γ), which is a crucial mediator of both innate and adaptive immune responses, is another cytokine that has recently been posited to play a role in stressor-related psychological pathology. Several studies have reported elevated circulating levels of IFN-γ among depressed patients (Simon et al., 2008; Gabbay et al., 2009; Dahl et al., 2014; Schmidt et al., 2014), and many of the most commonly used antidepressants were found to antagonize IFN-γ signaling (Maes et al., 1999; Kubera et al., 2001; Brustolim et al., 2006). Moreover, variation in the IFN-γ gene was recently reported to modify both depression risk (in the context of IFN-α treatment; Oxenkrug et al., 2011) and antidepressant medication effectiveness (Myint et al., 2013). Consistent with these findings, Kwant and Sakic (2004) reported that mice infected with IFN-γ adenovector displayed persistent anhedonic-like symptoms, and O’Connor et al. (2009a,b) showed that IFN-γ is a major driver of the indoleamine 2,3-dioxygenase (IDO)-enhancing and depressive-like behavioral effects of the immune-activating agents, lipopolysaccharide (LPS) and Bacillus Calmette–Guerin. Yet, compared with many of the other cytokines that have been linked to depressive illness, far fewer studies have actually set out to specifically test the influence of endogenous IFN-γ in ecologically inspired chronic stressor animal models; this is especially true in regards to the cognitive aspects of depressive-like pathology.

As one of the most potent activators of microglial cells and key regulator of the anti-viral response (Chesler and Reiss, 2002), IFN-γ is likely to be especially important for conditions in which infection overlaps with stressor exposure or in genetically vulnerable individuals (Litteljohn et al., 2010). In this regard, we previously found that IFN-γ-deficient mice had attenuated hormonal, cytokine and brain regional dopaminergic responses to chronic stress, despite showing several conspicuous behavioral and physiological differences in the basal state (i.e., increased anxiety-like behavior, elevated circulating corticosterone levels and central amygdala monoamine utilization; Litteljohn et al., 2010). This complex pattern of effects led us to theorize that IFN-γ contributes to a range of affective and perhaps cognitive processes, albeit probably in very different ways and to markedly different ends under basal and chronic stress conditions. Working under this theoretical framework, in the present investigation we sought to assess the largely unexplored role of IFN-γ in the spatial memory, psychomotor, and hippocampal BDNF and monoamine changes that are often evident following protracted exposure to psychologically relevant stressors.

## MATERIALS AND METHODS

### EXPERIMENTAL ANIMALS

Establishment of the IFN-γ –/– knockout (KO) mouse, which develops normally and is healthy in the absence of pathogenic challenge, has been described previously (Dalton et al., 1993). IFN-γ KO and wild-type (WT; IFN-γ +/+) mice raised on a C57BL/6J genetic background were obtained from The Jackson Laboratory (Bar Harbor, ME, USA) and interbred for several generations. At 6–7 weeks of age, KO and WT littermate controls from our breeding colony were singly housed in standard polycarbonate cages (27 cm × 21 cm × 14 cm). Animals were maintained on a 24 h light/dark cycle with lights on at 08:00. A diet of Ralston Purina (St. Louis, MO, USA) mouse chow and water was provided *ad libitum*, and room temperature was maintained at ∼21°C. All experimental procedures were approved by the Carleton University Committee for Animal Care and complied with the guidelines set out by the Canadian Council for the Use and Care of Animals in Research. The animals were between 10 and 12 weeks of age upon commencement of the study.

### CHRONIC STRESSOR REGIMEN

**FIGURE 1:**
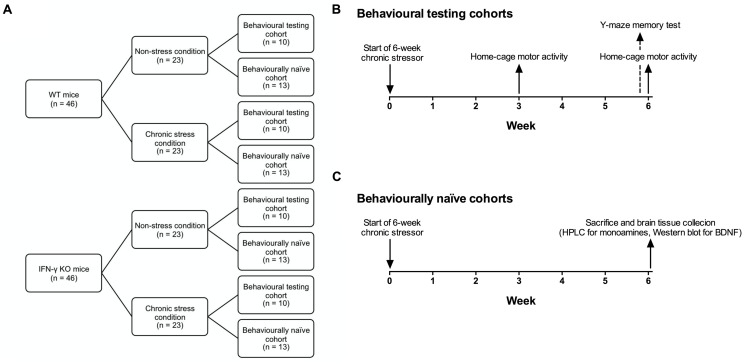
**Schematic showing the experimental design and timeline.** IFN-γ knockout (KO) and wild-type (WT) littermate mice (*n* = 46 per genotype) were randomly assigned to a 6-week chronic stressor condition or a non-stress control group (*n* = 23; **A**). Mice of each condition were further divided into behavioral testing (*n* = 10; **B**) and behaviorally naïve cohorts (*n* = 13; **C**), the latter of which were used for end-of-study brain regional monoamine (*n* = 10) or hippocampal BDNF analyses (*n* = 3).

**Figure 1** presents a schematic of the experimental design and timeline. Animals of either genotype (*n* = 46) were randomly assigned to a 6-week chronic stressor condition or a non-stress control group (*n* = 23). Mice of each condition were further divided into behavioral testing (*n* = 10) and behaviorally naïve cohorts (*n* = 13), the latter of which were used for end-of-study brain regional monoamine (*n* = 10) or hippocampal BDNF analyses (*n* = 3). The chronic stressor regimen comprised the application of two stressors per day (or a single stressor on behavioral testing days) on a variable and unpredictable schedule, and consisted of both mild and moderate stressors per the method and rationale of Litteljohn et al. (2010). A list of the various stressors used is provided in **Table 1**. Following the first (morning) stressor, mice were returned to their home-cage until application of the second (afternoon) stressor. Animals assigned to the chronic stressor condition were housed in a holding room separate from, but otherwise identical to, their non-stressed counterparts.

**Table 1:** List of stressors.

Stressor	Duration	Specifications
Social interaction	60 min	Placement in a large cage (40 cm × 25 cm × 15 cm) divided into separate quadrants with three non-experimental male C57BL/6J mice (3–6 months old); this set-up allowed for interactions but not fighting
Soiled cage	60 min	Introduction into a congener’s soiled cage
Fox urine	5 min	Exposure to 250 cc fox urine-infested air (Foxpert, St. Benjamin, QC, Canada) while in a novel, empty cage
Rat feces	60 min	Introduction into an unfamiliar cage with fresh rat feces
Flat bottom restraint	15 min	Restraint in semicircular Plexiglas tubes (4 cm × 12 cm) with tails taped to prevent mice from turning
Plastic bag restraint	15 min	Restraint in tight-fitting triangular plastic bags equipped with a nose-hole for breathing
Footshock	—	15 shocks, 500 ms duration at 30 s intervals, 0.3 mA, 60 Hz, a.c.) administered in individual shock chambers (30 cm × 14 cm × 15 cm)
Injection/handling	—	Intraperitoneal injection of 0.2 ml sterile physiological saline (Sigma Aldrich, USA)
Damp bedding	60 min	60 ml of water/l of sawdust bedding in novel cage
Tail hang	30 s	—
Empty cage	60 min	Introduction into an empty cage without sawdust or nestlet
Noise	10 min	Intermittent background noise (40 dB) in isolated restraint chambers (30 cm × 14 cm × 15 cm)
Cage tilt	60 min	30° tilt of home-cage
Forced swim	3 min	Forced swim in a glass cylinder (20 cm diameter × 25 cm high) containing tepid water (22 ± 1°C, 15 cm deep)
Light/dark cycle disruption	12 h	Lights on during dark phase

### BEHAVIORAL ASSESSMENTS

#### Spontaneous home-cage activity

Measurements of horizontal motor activity were obtained during complete, uninterrupted 12 h light/dark cycles using a Micromax infrared beam-break apparatus (AccuScan Instruments, Columbus, OH, USA) exterior to the home-cage. The same animals were tested on two separate occasions, corresponding to the midway point (Week 3: Day 21) and endpoint (Week 6: Day 42) of the stressor paradigm, and the data analyzed using a repeated measures analysis of variance (ANOVA). Testing commenced 60 min after termination of the morning stressor.

#### Spatial memory in the two-trial Y-maze

During the final week of the experiment (Day 40), intermediate-term spatial recognition memory was assessed in a two-trial Y-maze task, in accordance with previously published methods (Dellu et al., 2000; Ferguson et al., 2000). The testing apparatus comprised three arms (30 cm × 8 cm × 15 cm) fashioned from black Plexiglas with an outer wood shell. The testing room was dimly lit and had on its walls various cardboard cut-outs of basic geometric shapes. During the first trial (acquisition phase) one arm of the maze was blocked with an opaque, removable panel. Mice were then placed individually in one of the two remaining accessible arms (i.e., the ‘start’ arm, which remained so in the second trial), with head directed away from the center of the maze. Animals were allowed to explore the open arms of the Y-maze for 5 min, after which they were returned to the home-cage. After 30 min, the second trial (retrieval phase) was conducted under identical experimental conditions to the first, excepting that the mice were now permitted free exploration for 5 min of all three arms of the maze (start, familiar and novel). The blocked arm (i.e., novel arm in the retrieval phase) varied between mice in a predetermined, pseudo-random manner, and the maze was cleaned with a dilute (2%) ethanol solution after each trial. Total arm entries (for each trial, defined as all four legs having entered a given arm) and % duration in the novel, start and familiar arms (Trial 2) were determined for each mouse. A discrimination index (DI) for novelty was calculated as follows: novel arm duration - (start arm duration + familiar arm duration)/2. A DI value not significantly different than zero (0) is understood to reflect a deficit in novelty discrimination (e.g., see Leconte et al., 2011). Y-maze testing occurred between 09:00 and 13:00 (i.e., 17–21 h after the previous day’s afternoon stressor), after which mice in the stressor groups received a single afternoon stressor.

### BRAIN DISSECTION METHOD

Animals were rapidly decapitated between 09:00 and 11:00 on the day following the completion of the 6-week chronic stressor regimen (i.e., 17–19 h after the final stressor treatment, which for all animals was a 15-min flat-bottom restraint). Brains were excised and sectioned into sequential coronal slices using razor blades and a chilled stainless steel microdissecting block with adjacent slots arranged 0.5 mm apart. The locus coeruleus and nucleus accumbens were obtained by micropunch using a hollow 1.0 mm diameter biopsy needle (collected bilaterally), whereas the dorsal hippocampus was microdissected in its entirety using chilled razor blades. Brain tissue samples were taken with reference to the mouse brain atlas of Franklin and Paxinos (1997). A subset of the hippocampal samples was flash-frozen and stored at -80°C for later determination of BDNF content by Western blot (*n* = 3). The remaining samples were maintained in a homogenizing solution containing 14.17 g monochloroacetic acid, 0.0186 g EDTA, 5.0 ml methanol, and 500 ml high performance liquid chromatography (HPLC) grade water; and stored at -80°C for HPLC analysis.

### HPLC DETERMINATION OF CENTRAL AMINE AND METABOLITE CONCENTRATIONS

Levels of norepinephrine (NE), serotonin (5-HT) and dopamine (DA), and their respective primary metabolites, 3-methoxy-4-hydroxyphenylglycol (MHPG), 5-hydroxyindole acetic acid (5-HIAA), and 3,4-dihydroxyphenylacetic acid (DOPAC) and homovanillic acid (HVA), were determined by HPLC within relevant brain punches according to previously reported methods (Liu et al., 2014). Tissue punches were homogenized by ultrasonic disruption (Sonic Dismembrator Model 100, Fisher Scientific) in the homogenizing solution in which they were initially frozen (with DHBA as an internal standard). The level of protein was determined with the Pierce BCA Protein Assay Kit (Thermo Scientific 23225). Homogenized samples were centrifuged (12000 rpm for 3 min at 4°C), after which 50 μl of supernatant was injected, at a flow rate of 1 ml/min, into the automated HPLC system (Agilent 1100) with electrochemical detector (DECADE II SDC, Antec) and ZORBAX Eclipse XDB-C8 columns (Agilent: 4.6 mm inner diameter, 150 mm length, 5 μm particle size; thermostated at 40°C); the oxidation potential was maintained at 0.60 V. The mobile phase comprised: 90 mM sodium phosphate monobasic, 1.7 mM 1-octanesulfonic acid, 50 mM EDTA, 10% acetonitrile, 50 mM citric acid (monohydrate), 5 mM KCL, and HPLC-grade water. Monoamine and metabolite concentrations were expressed relative to the protein content of the samples, and final results presented as ng/mg protein.

### HIPPOCAMPAL BDNF PROTEIN DETERMINATION

Western immunoblotting was performed largely in accordance with our previously published methods (Mangano et al., 2011). Briefly, brain tissues (*n* = 3) were homogenized on ice in RIPA lysis buffer containing 50 mM Tris-base (pH 8.0), 150 mM NaCl, 1% Triton-X, 0.1% SDS, 0.5% sodium deoxycholate, and cOmplete Mini EDTA-free protease inhibitor (Roche, Basel, Switzerland). Lysates were centrifuged for 5 min (5000 rpm at 4°C) and supernatants collected. Total protein was then determined using a BCA Protein Assay kit (Thermo Fisher Scientific, Waltham, MA, USA). Protein from the hippocampus (50 μg/well) was diluted to a final volume of 35 μl in RIPA lysis buffer and 1X loading buffer (5% glycerol, 5% β-mercaptoethanol, 3% SDS, and 0.05% bromophenol blue), and samples heated in boiling water for 5 min. Proteins were separated by electrophoresis (120 V) on 12.5% sodium dodecyl sulfate-polyacrylamide gels and transferred overnight at 4°C (180 mA) onto PVDF membranes (Bio-Rad, Hercules, CA, USA). Membranes were then blocked for 1-h with gentle agitation at room temperature in a Tris-buffered saline [TBS-T: 10 mM Tris-base (pH 8.0), 150 mM NaCl, 0.5% Tween-20] solution containing non-fat dry milk (5% w/v). Anti-BDNF primary antibody (1:500, sc-546, Santa Cruz Biotechnology, Dallas, TX, USA) was applied for 1.5-h at room temperature. After four successive 10-min washes in TBS-T, membranes were incubated with secondary antibody for 1-h at room temperature and with gentle shaking (goat anti-rabbit IgG peroxidase, 1:1000, A6154, Sigma). After another series of TBS-T washes, bands were visualized by exposing Kodak X-OMAT film (10 min for BDNF, 10 s for β-actin) to membranes treated with ECL substrate (Perkin Elmer, Waltham, MA, USA; for 1 min). The immunoblots were imaged using a Konica Minolta SRX-101A processor (Konica Minolta, Marunouchi, Chiyoda-ku, Tokyo), and band density quantified using AlphaEaseFC v.3.1.2 densitometry software (Alpha Innotech, San Leandro, CA, USA). After normalizing against β-actin (anti-β-actin; 1:5000, sc-47778, Santa Cruz), the BDNF/actin ratios were averaged across blots and the standard error of the mean determined for each treatment group.

### STATISTICAL ANALYSES

The monoamine and Western immunoblot data were analyzed by 2 (Genotype; WT vs. IFN-γ KO) × 2 (Treatment; non-stressed vs. stressed) ANOVAs followed where appropriate by Student–Newman–Keuls pairwise multiple comparisons (*p* < 0.05). The home-cage activity data were analyzed by a repeated measures ANOVA with Genotype and Treatment as the between-subjects variables and Time (Week 3 vs. Week 6) as the repeated measures variable. A mixed model ANOVA was also used for analyzing % duration in the three arms of the Y-maze; here, Genotype and Treatment were the between-subjects variables and Arm (novel vs. start vs. other) served as the within-subjects variable. Planned univariate *t*-tests facilitated comparisons of spatial memory performance in each of the experimental groups (% novel arm duration, DI) with that of a theoretical group performing at chance-level (33.33% and 0, respectively). In addition, Spearman’s rank-order correlation coefficients (ρ) were calculated to assess the degree of association between locomotion (total arm entries) and memory performance (DI) in the Y-maze; separate analyses were conducted for the total sample (collapsing across IFN-γ KO and stress) and each of the four treatment groups. On account of procedural error, one mouse was excluded from the home-cage activity assessments and two mice excluded from the Y-maze analyses. During the course of tissue dissection and monoamine determination a few samples were lost due to error or variability (>2.5 standard deviation from the mean); hence, the degrees of freedom for the statistical analyses varied within and across some brain regions and/or neurochemical substrates. Data were evaluated using a StatView (version 6.0) statistical software package and plotted with GraphPad Prism 6 (La Jolla, CA, USA).

## RESULTS

### CHRONIC STRESSOR TREATMENT TIME-DEPENDENTLY INFLUENCED HOME-CAGE ACTIVITY IN IFN-γ WILD-TYPE AND KNOCKOUT MICE

**FIGURE 2:**
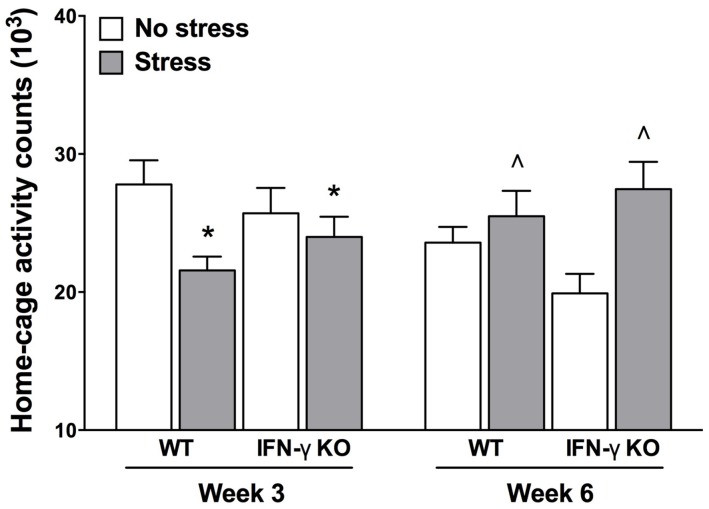
**Influence of chronic stress on home-cage locomotor activity among IFN-γ wild-type and knockout mice.** Overall, chronic stressor exposure had the effect of reducing home-cage activity at Week 3 (mid-way through the experiment), but increasing locomotor activity at Week 6 (end of experiment). However, the early occurring hypolocomotive effect of stress was clearly most prominent in the WT animals, whereas the stressor’s later-occurring hyperlocomotive effect was most evident in the KO mice (see accompanying text). Data are presented as mean ± SEM. *p < 0.05 relative to non-stressed mice (collapsed across genotype) at Week 3, and ˆ*p* < 0.05 relative to non-stressed mice (collapsed across genotype) at Week 6 (Two-way repeated measures ANOVA). WT, wild-type; KO, knockout.

It is understood that psychologically relevant stressors can modulate motor functioning, and sometimes in diametrically opposed ways (Soblosky and Thurmond, 1986; Venzala et al., 2012). Indeed, psychomotor symptoms are a quite common occurrence in depression, as well as myriad other stressor-related psychiatric conditions (Buyukdura et al., 2011). Here, we report the outcomes of our time series analysis of home-cage locomotor activity. The repeated measures ANOVA revealed significant Genotype × Treatment and Treatment × Time interactions for home-cage activity (*F*s_1,35_ = 5.27 and 14.66, respectively, *p* < 0.05). As shown in **Figure 2**, while at the midway point of the experiment (Week 3) motor activity was significantly reduced by the chronic stressor, by the end of the experiment (Week 6) animals that were exposed to the stressor actually displayed increased home-cage activity (*p* < 0.05). Despite finding a significant Genotype × Treatment interaction for home-cage activity, follow-up analyses failed to reveal any statistically significant simple main effects. The existence of such a “crossover” interaction suggests that the early occurring, hypolocomotive effect of the chronic stressor predominated in the WT animals, whereas the later-occurring, activity-boosting effect of the stressor was most pronounced in the KOs; this interpretation is borne out by visual inspection of the data (see **Figure 2**). Moreover, the multiple Bonferroni-corrected pairwise comparisons revealed that, at Week 6, only the stressed IFN-γ KO mice displayed significantly higher levels of activity compared to their non-stressed counterparts (*p* < 0.0018).

### CHRONIC STRESS FACILITATED SPATIAL RECOGNITION MEMORY IN THE IFN-γ KNOCKOUT MICE

**Table 2** presents the total number of arm entries during the acquisition (Trial 1) and retention phases (Trial 2) of the Y-maze test. During the acquisition phase, the IFN-γ KOs made fewer total arm entries than the WT animals, regardless of stressor treatment (*F*_1,34_ = 9.05, *p* < 0.01). In contrast, during the retention phase the total number of arm entries did not differ significantly between groups (*F*s < 2.8, see **Table 2**). Since the acquisition phase of the Y-maze task is comparatively anxiety-laden, it is not surprising that the IFN-γ KOs, for which we and others have previously described an anxious phenotype (Kustova et al., 1998; Litteljohn et al., 2010; Campos et al., 2014), should display reduced activity in this context.

**Table 2:** Total number of arm entries during the Y-maze acquisition and retention phases.

	Treatment condition			
	WT-No stress	WT-stress	KO-no stress	KO-stress
Acquisition phase	27.4 ± 1.1	25.1 ± 2.5	21.1 ± 1.4*	20.7 ± 1.5*
Retention phase	25.1 ± 1.8	20.1 ± 1.6	21.1 ± 1.7	20.6 ± 1.5

Data are presented as mean ± SEM (*n* = 8–10). *p < 0.05 relative to wild-type mice (collapsed across the stressor treatment). WT, wild-type; KO, knockout.

**FIGURE 3:**
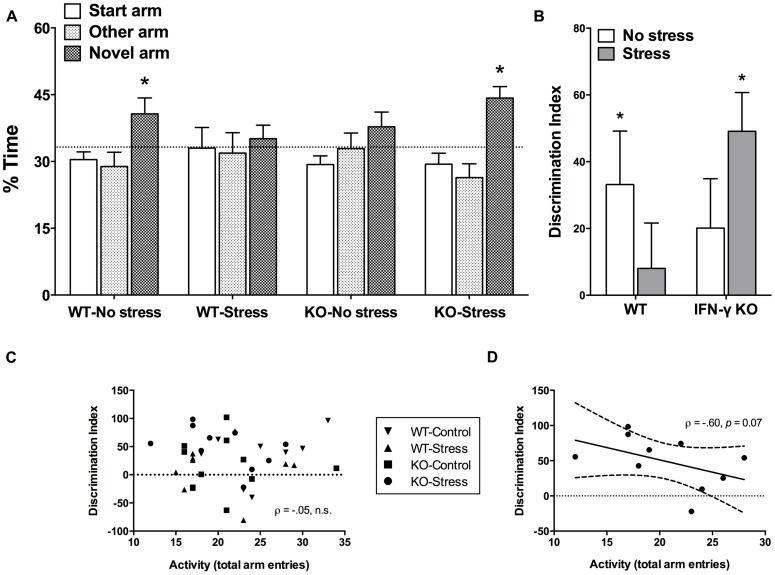
**Impact of IFN-γ deletion on short-term spatial memory function under basal vs. chronic stressor conditions.** As regards both the percentage (%) time spent exploring the novel arm of the Y-maze **(A)** and the novelty discrimination index (DI; **B)**, of the four treatment groups, only the non-stressed wild-type controls and the chronically stressed IFN-γ knockout mice performed at a level significantly above chance (33.3% and 0, respectively). While overall there was no association between DI scores and total Y-maze arm entries **(C)**, when the correlation analysis was restricted to the chronically stressed knockouts, Y-maze locomotion tended to correlate negatively with spatial memory performance **(D**; see accompanying text). Data are presented as mean ± SEM; horizontal dashed lines represent chance-level performance (33.3% or 0), the solid line represents the best fit linear regression line, and the curved dashed lines show the 95% confidence interval. *p < 0.05 relative to chance (univariate *t*-test). WT, wild-type; KO, knockout.

Time spent exploring the novel arm of the Y-maze is considered a reliable index of spatial memory functioning in rodents (Dellu et al., 2000; Leconte et al., 2011). The initial mixed model ANOVA revealed a significant main effect of Arm (*F*_2,68_ = 6.88, *p* < 0.01), such that overall the mice spent significantly more time exploring the novel vs. start or familiar arms (*p* < 0.05). Yet, as shown in **Figure 3A** and confirmed by the planned univariate *t*-tests, of the four treatment groups, only the non-stressed WT controls and the chronically stressed IFN-γ KO mice performed significantly above chance-level (33.33%, *p* < 0.05). Equivalently, our analysis of DI scores revealed that whereas both the WT control and stressed KO animals discriminated the novel arm to a significant extent (DI scores greater than 0, *p* < 0.05, see **Figure 3B**), neither the non-stressed IFN-γ KO controls nor the chronically stressed WT mice had DI values significantly different than 0 (*p*> 0.1). However, the corresponding ANOVA failed to uncover any statistically significant between-group differences (*F*s < 3.75), and the within-group variability of DI scores was clearly quite considerable (see **Figure 3B**).

In view of the stressor-induced and genotype-specific changes in home-cage activity, and despite the lack of significant treatment effects on total arm entries in the retention phase of the Y-maze, it was of interest to determine whether Y-maze locomotion correlated with spatial memory performance. In this regard, there was an utter lack of association between DI scores and total Y-maze arm entries when collapsing across the treatment groups (ρ = –0.05, *p* > 0.70, see **Figure 3C**). This was similarly the case when separate analyses were performed for the WT control, WT stressed and IFN-γ KO control groups (ρ = 0.43, 0.14, -0.14, respectively, *p* > 0.25). Yet, in the case of the chronically stressed KOs, though the negative correlation between DI scores and Y-maze activity was not statistically significant at the α = 0.05 level (ρ = -0.60, *p* = 0.07), the *p* value was considerably less than 0.10. While acknowledging the need for caution in interpreting this marginally significant trend, as seen in **Figure 3D** it would appear that, in the chronically stressed KOs only, reduced Y-maze locomotion tended to correspond with better spatial memory performance.

### BRAIN REGIONAL MONOAMINERGIC EFFECTS OF CHRONIC STRESS AND IFN-γ KNOCKOUT

**FIGURE 4:**
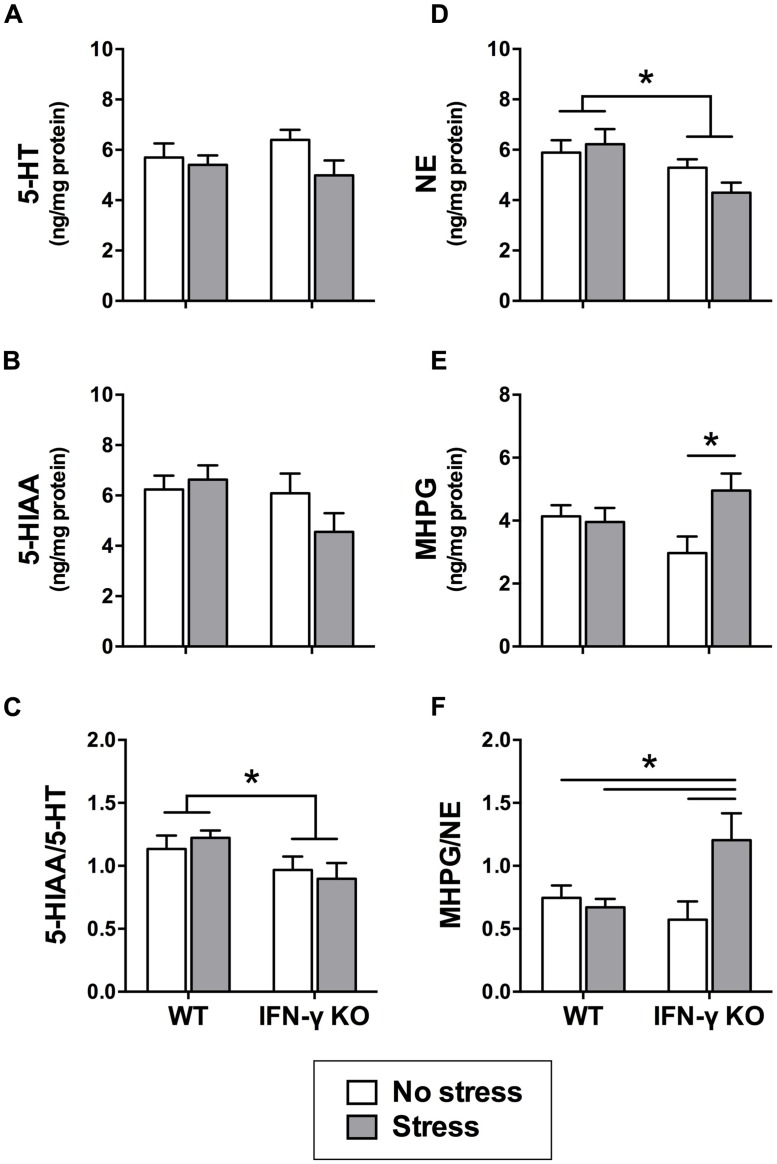
**Hippocampal serotonin and noradrenergic activity as a function of IFN-γ knockout and chronic stressor exposure.** Within the hippocampus, neither 5-HT **(A)** nor 5-HIAA **(B)** was significantly affected by chronic stress or IFN-γ deletion. Yet, 5-HT turnover (i.e., the ratio of 5-HIAA to 5-HT; **C)** was diminished overall in the IFN-γ knockouts compared to the wild-type mice. With respect to noradrenergic neurotransmission in the hippocampus, NE levels **(D)** were significantly reduced among the IFN-γ knockout mice, irrespective of chronic stress. In contrast, both MHPG accumulation **(E)** and NE turnover (MHPG-to-NE ratio; **F)** were significantly enhanced following chronic stress, but only among the IFN-γ knockouts. Data are presented as mean ± SEM; *p < 0.05 (Two-way ANOVA followed by Student–Newman–Keuls test). WT, wild-type, KO, knockout.

Within the dorsal hippocampus, neither chronic stress nor IFN-γ deletion significantly affected the levels of 5-HT or its primary metabolite, 5-HIAA (*F*s < 2.7; see **Figures 4A,B**). However, as shown in **Figure 4C**, 5-HT turnover (i.e., the ratio of metabolite to parent amine) was significantly diminished overall among mice genetically lacking IFN-γ (*F*_1,34_ = 5.75, *p* < 0.05). A separate ANOVA revealed that the KOs also had diminished hippocampal NE levels relative to the WT mice, regardless of stressor history (*F*_1,33_ = 7.15, *p* < 0.05; see **Figure 4D**). In addition, concentrations of the primary NE metabolite, MHPG, varied according to the significant interaction of Genotype with Stress (*F*_1,33_ = 5.05, *p* < 0.05). As shown in **Figure 4E** and confirmed by the *post hoc* comparisons, whereas chronic stress had no effect on hippocampal MHPG levels in the WT animals, accumulation of the metabolite was robustly enhanced in the stressed KOs (*p* < 0.05, relative to KO controls). A significant Genotype × Stress interaction was likewise uncovered for NE turnover (*F*_1,32_ = 6.24, *p* < 0.05) such that the ratio of hippocampal MHPG to NE was markedly elevated in the stressed IFN-γ KOs compared to all other groups (*p* < 0.05; see **Figure 4F**).

**FIGURE 5:**
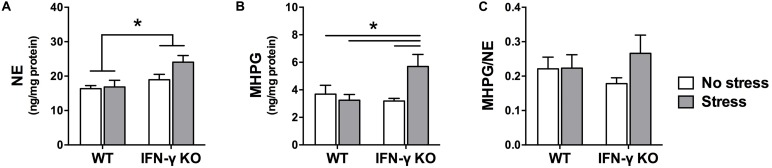
**Locus coeruleus noradrenergic activity as a function of IFN-γ knockout and chronic stressor exposure.** Compared to the wild-type animals, concentrations of locus coeruleus NE **(A)** were significantly higher among the IFN-γ-deficient mice, regardless of stressor exposure. As regards MHPG in this brain region **(B)**, whereas metabolite levels were unaffected by chronic stress in the wild-type mice, among the stressed IFN-γ knockouts MHPG accumulation was significantly enhanced. The experimental manipulations did not, however, significantly alter locus coeruleus NE turnover (ratio of MHPG-to-NE; **C)**. Data are presented as mean ± SEM; *p < 0.05 (Two-way ANOVA followed by Student–Newman–Keuls test).

Within the locus coeruleus, NE concentrations were significantly higher in the IFN-γ-deficient animals compared to their WT littermates, irrespective of chronic stressor exposure (*F*_1,35_ = 8.48, *p* < 0.01; see **Figure 5A**). But akin to what was observed in the hippocampus, locus coeruleus MHPG levels varied as a function of the interaction between Genotype and Stress (*F*_1,34_ = 6.18, *p* < 0.05). As depicted in **Figure 5B** and confirmed by the follow-up tests, among the IFN-γ KOs chronic stress induced a marked rise in MHPG concentrations (*p* < 0.05 compared to all other groups). In WT mice, however, MHPG levels were completely unaffected by the stressor. Notwithstanding these changes, the ANOVA for locus coeruleus NE turnover did not reveal any significant main or interaction effects of IFN-γ deletion and chronic stress (*F*s < 1.4; see **Figure 5C**).

Dopaminergic gating of information through the stressor-sensitive nucleus accumbens is considered to play an important role in both motor and memory function (Costall et al., 1984; Mele et al., 2004; Baker and Kalivas, 2005). It was therefore of interest in the present study to characterize the accumbal dopaminergic effects of chronic stress and IFN-γ deficiency. As shown in **Table 3**, neither of the experimental treatments (nor their interaction) significantly affected indices of dopaminergic neurotransmission in this brain region (DA, DOPAC, and HVA concentrations, as well as DA turnover; *F*s < 3.4).

**Table 3:** Dopaminergic activity within the nucleus accumbens as a function of chronic stress and IFN-γ deletion.

	Concentration (ng/mg protein)			DA turnover [(DOPAC+HVA)/DA]
	DA	DOPAC	HVA	
WT-no stress	123.57 ± 14.76	15.43 ± 1.19	7.95 ± .43	0.216 ± 0.035
WT-stress	152.78 ± 17.56	15.98 ± 1.04	5.96 ± .91	0.173 ± 0.039
KO-no stress	156.33 ± 20.07	16.81 ± 1.36	5.50 ± .54	0.155 ± 0.017
KO-stress	186.87 ± 19.00	17.67 ± 1.61	6.28 ± .85	0.145 ± 0.022

*Data are presented as mean ± SEM. DA, dopamine; DOPAC, 3,4-dihydroxyphenylacetic acid; HVA, homovanillic acid.*

### HIPPOCAMPAL BDNF EXPRESSION WAS UNCHANGED FOLLOWING CHRONIC STRESSOR EXPOSURE

**FIGURE 6:**
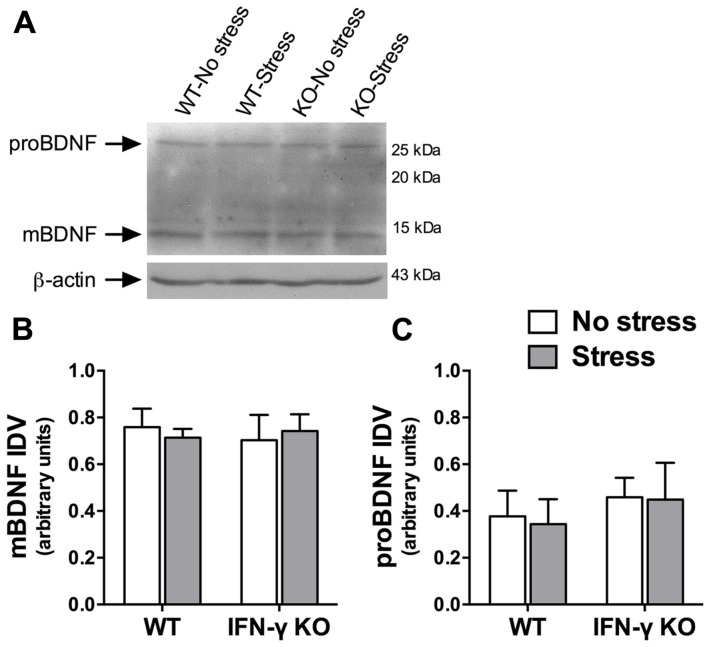
**Influence of chronic stress and IFN-γ knockout on BDNF protein expression within the hippocampus.** Representative immunoblots **(A)** were cropped and adjusted for brightness and contrast using Fotor photo editor software (version 1.3.1). Neither chronic stress nor IFN-γ knockout significantly affected mature BDNF **(B)** or proBDNF **(C)** protein expression within the hippocampus. Actin-normalized integrated density values (IDVs) are presented as mean ± SEM (*n* = 3).

A large body of evidence demonstrates that the neurotrophic factor, BDNF, is essential for hippocampus-dependent memory function and adaptive neuroplastic responses to stressors (Schmidt and Duman, 2010; Taliaz et al., 2010). We were therefore somewhat surprised to find that neither chronic stress nor IFN-γ deletion significantly influenced hippocampal BDNF protein expression (*F*s < 1, see **Figures 6A,B**). The Western immunoblot analysis likewise failed to reveal any significant main or interaction effects of the experimental treatments on the hippocampal protein concentrations of a neuronally secreted immature form of BDNF (i.e., proBDNF; *F*s < 1, see **Figures 6A,C**).

## DISCUSSION

Accumulating evidence suggests a role of IFN-γ in depression and other stressor-associated psychological disturbances (O’Connor et al., 2009a; Dahl et al., 2014); however, the question whether IFN-γ contributes to the pathological process in ecologically inspired chronic stressor models has gone largely untested. In what is to the best of our knowledge the only other study published to date on this subject, we previously reported that IFN-γ deficiency conferred protection in mice against some of the immune, stress hormone and limbic monoamine effects associated with chronic exposure to a psychologically relevant stressor (Litteljohn et al., 2010). Intriguingly, our results also suggested that IFN-γ may have divergent emotion-relevant actions under normal and chronic stressor conditions, as the KO animals basally showed enhanced anxiety-like behavior coupled with heightened corticosteroid levels and central amygdala NE and 5-HT usage. Such findings are actually consistent with the earlier results of Kustova et al. (1998), as well as the more recent ones of Campos et al. (2014), which indicated that mice genetically lacking IFN-γ are characterized basally by increased emotionality and anxiety. The results of the present investigation extend these findings by demonstrating that stressor context also appears to be crucial in determining the influence of IFN-γ on spatial memory function and related neurochemical systems. Indeed, under normal conditions mice genetically lacking IFN-γ exhibited impaired spatial memory, which we suggest might be related to altered hippocampal (and perhaps locus coeruleus) monoaminergic neurotransmission but not BDNF signaling or nucleus accumbens dopaminergic activity. Contrastingly, under conditions of chronic stress IFN-γ deficiency appeared actually to facilitate memory function, and this pro-mnemonic effect coincided with enhanced hippocampal and locus coeruleus noradrenergic activity.

### IMPACT OF IFN-γ DEFICIENCY ON MEMORY AND RELATED BRAIN PROCESSES UNDER NORMAL CONDITIONS

It is now well recognized that pro-inflammatory cytokines can modulate cognitive processes, with pathological consequences probably at the forefront of attention. Yet, there is increasing evidence to suggest that cytokines and other pro-inflammatory stimuli (e.g., prostaglandins, amyloid-β peptide: Cowley et al., 2008; Puzzo et al., 2012) may under certain circumstances actually be beneficial for learning and memory; the key determinant here appears to be cytokine level (McAfoose and Baune, 2009; Yirmiya and Goshen, 2011). While highly elevated concentrations of pro-inflammatory cytokines, as can occur for instance in depression and many of its comorbid conditions (Anisman and Hayley, 2012; Dahl et al., 2014), generally provoke anti-mnemonic effects, it would appear that a certain basal physiological level of pro-inflammatory cytokine signal is required for normal memory function (Yirmiya and Goshen, 2011). Evidencing the latter, mice genetically lacking TNF-α or TNF receptor 1 displayed impaired spatial memory (TNF-R2 KO mice, however, displayed intact memory; Camara et al., 2013), and IL-1β signaling blockade produced a similar functional effect (Yirmiya et al., 2002; Goshen et al., 2007).

Consistent with such a view, we presently report that mice lacking IFN-γ showed disturbed spatial recognition memory in the basal state. Interestingly, Baron et al. (2008) revealed that *limited* central overexpression of IFN-γ resulted in improved hippocampus-dependent memory, whereas pathologically elevated concentrations of the cytokine have generally been associated with memory impairment (Lapter et al., 2009; Dutra et al., 2013; Too et al., 2014). Thus, when considered together, these data suggest that the hormetic-like dose-response pattern that was described elsewhere for memory and IL-1β (and several other immune actors; Yirmiya and Goshen, 2011) may very well be relevant too for IFN-γ. Further investigation is warranted to substantiate this possibility, and such efforts will do well to include a detailed time-and-dose-response analysis, as well as determinations of both circulating and brain regional IFN-γ concentrations in ecologically relevant animal disease models. Also, some caution should be exercised when interpreting the Y-maze behavioral data in the present study: while basally the KOs failed to perform significantly better than chance (consistent with impaired spatial memory), the ANOVA test did not reveal any significant between-group differences. As mentioned previously, there was considerable within-group variability in the behavioral data, and this likely reflects the critical but often overlooked influence of individual differences in chronic stress susceptibility (e.g., Bergström et al., 2008). Therefore, it is our suggestion that the present findings be viewed as proof-of-principle for more comprehensive and larger-scale investigation into the prospective learning and memory effects of IFN-γ.

With respect to the possible neural substrate(s) subserving these memory changes, we submit that it may be particularly telling that the IFN-γ KOs overall displayed altered NE content within both the locus coeruleus and hippocampus (increased in the former brain region and reduced in the latter), as well as diminished hippocampal serotonin turnover. Each of these highly interconnected, stressor-sensitive brain regions plays a vital role in memory function, with the hippocampus considered especially critical for memory consolidation and spatial navigation (McGaugh, 2000; Suzuki, 2006), and the noradrenergic locus coeruleus mediating the potent cognition-modulatory effects of emotional arousal (Gibbs et al., 2010; Sara and Bouret, 2012). Moreover, human and animal studies alike have implicated both noradrenergic and serotonergic signaling in the memory process, and generally in a facilitatory capacity (Lee and Ma, 1995; Murchison et al., 2004; Meeter et al., 2006). It seems reasonable therefore to suggest that dysregulated monoaminergic neurotransmission, particularly in the hippocampus, could have been at least partially responsible for the memory deficit seen in these animals. Notably, memory impairment may have occurred both in spite of the observed increase of locus coeruleus NE (e.g., Gibbs et al., 2010) and independently of dopaminergic neurotransmission in the nucleus accumbens (which as will be recalled was unaffected by the experimental manipulations). Importantly, since the KO-specific hippocampal and locus coeruleus monoamine changes occurred irrespective of stressor challenge, it would appear that IFN-γ normally plays a role in the homeostatic regulation of these neurotransmitter systems (but presumably not the accumbal DA system). Alternatively, as the KOs were without IFN-γ signal from birth, it is possible that physiological concentrations of the cytokine are required for the normal maturation of these monoamine systems across development (Litteljohn et al., 2010).

Of course, such a reading cannot discount the possibility that other molecular and/or cellular processes might also have contributed to the presently described memory effects. Indeed, in our previous work (Litteljohn et al., 2010) we showed that mice genetically lacking IFN-γ had increased basal corticosterone levels and central amygdala monoamine activity, and we argued that such changes were relevant to the anxious phenotype already on record for these animals (Kustova et al., 1998). Yet, hippocampus-dependent memory is also subject to modulation by corticosterone and central amygdala monoaminergic neurotransmission – mainly retrieval in the case of the former and consolidation as regards the latter (de Quervain et al., 1998; Hermans et al., 2014) – and it is entirely plausible that one or both of these processes could have contributed to basal memory impairment among the IFN-γ KOs. Similarly, as a wealth of evidence has implicated hippocampal BDNF in memory function, with a reduction of neurotophin levels generally being tied to poor memory outcomes and an increase in BDNF levels usually signaling the opposite (Mizuno et al., 2000; Shirayama et al., 2002), we had speculated that any IFN-γ KO-associated decline in memory might also be attended by a reduction in hippocampal BDNF. Contrary to our expectations, however, it will be recalled that neither mature nor proBDNF protein levels were affected by IFN-γ deletion. These null BDNF results are in agreement with the recent enzyme-linked immunosorbent assay (ELISA)-based findings of Campos et al. (2014), and together our studies provide good evidence that altered hippocampal BDNF does not underlie the neurobehavioral phenotype of IFN-γ KO mice. That said, our results do not preclude the involvement of BDNF in prospective IFN-γ-associated proactive memory effects; in fact, Baron et al. (2008) provide evidence that the memory enhancement seen in mice overexpressing IFN-γ may at least partially be attributable to an upregulation of central BDNF.

### INFLUENCE OF IFN-γ KNOCKOUT ON MEMORY AND RELATED NEUROCHEMICAL PROCESSES UNDER CHRONIC STRESSOR CONDITIONS

If a lack of IFN-γ can under normal conditions be seen to predispose to memory dysfunction, under conditions of chronic stress the result seems to be memory facilitation. Indeed, whereas neither the basal state KOs nor the chronically stressed WT mice demonstrated Y-maze performance that was significantly better than chance, both the stressor-treated IFN-γ-deficient mice and the non-stressed WT controls displayed intact spatial memory. These data are aligned somewhat with the findings of other studies indicating that chronic variable stress can paradoxically (and akin to what’s been reported for acute as well as predictable chronic stress: e.g., Parihar et al., 2011; Uysal et al., 2012) enhance memory and learning (Bartolomucci et al., 2002; McLaughlin et al., 2005; Hawley et al., 2012); here we provide evidence suggesting that IFN-γ, or rather a lack thereof, may be key. Interestingly, paralleling the behavioral data, our neurochemical analyses revealed that noradrenergic, but not serotonergic, metabolism was markedly and selectively augmented in the hippocampus and locus coeruleus among IFN-γ null mice that were exposed to the chronic stressor. Given the aforementioned importance of these brain regions in learning and memory (Gibbs et al., 2010), and the generally facilitative role ascribed to noradrenergic signaling in this regard (Lee and Ma, 1995), it appears likely that IFN-γ acts to restrict brain noradrenergic and, *consequently*, spatial memory responses to chronic stress.

Recent reports have documented elevations of circulating and brain IFN-γ levels among rodents exposed to chronic stressors (Liu et al., 2013; Wrona et al., 2014). And while these findings contrast with those of several other animal (e.g., Palumbo et al., 2012) as well as human studies (Segerstrom and Miller, 2004), there is a growing recognition that stress hormones can under certain circumstances augment brain inflammation (Sorrells et al., 2009). A number of routes exist by which IFN-γ could come to be influenced by, and hence contribute to, the central actions of psychological stressors (Litteljohn et al., 2010). One such potential mechanism involves a stressor-induced shift in the T-helper type-1 (Th1)/T-helper type-2 (Th2) cytokine balance in favor of the former (though stressor chronicity is a major influence here: Maes et al., 1998; Segerstrom and Miller, 2004). In this way, depression related pro-inflammatory Th1 responses (e.g., IDO activation and 5-HT depletion), of which IFN-γ is the principal effector, could become accentuated at the expense of anti-inflammatory Th2 ones (e.g., those mediated by IL-4 and IL-10; Najjar et al., 2013). Stressors can also provoke intestinal barrier dysfunction and mucosal inflammation (Vicario et al., 2010), which could lead to not only increased circulating and even central IFN-γ levels but also the potentiated trafficking of immune cells into the brain parenchyma (Tran et al., 2000; Schroder et al., 2004). Notably, the latter could also be realized through a stressor-induced disruption of blood–brain barrier integrity (Friedman et al., 1996; Northrop and Yamamoto, 2012). And finally, that brain-resident microglia are themselves capable of producing IFN-γ under the direction of endogenous cytokine signals (i.e., emanating from other glial cells and not necessarily brain-infiltrating leukocytes; Kawanokuchi et al., 2006) raises the intriguing possibility that stressors could act directly on microglial cells to influence central IFN-γ signaling.

Yet, it should be noted that by the end of the 6-week stressor regimen the chronically stressed KOs also displayed increased spontaneous locomotor activity; this was evident too in the stressed WT animals, but seemingly to a lesser degree. Several studies have linked hyperactivity in rodents to increased brainstem and hippocampal noradrenergic activity (though the role of NE in regulating motor brain circuitry is not straightforward and almost certainly involves cross-talk with brain DA and 5-HT systems; Suwabe et al., 2000; Ruocco et al., 2010; Lambertsen et al., 2012), and there is reason to think that a similar situation could be relevant to certain clinical contexts – for instance ADHD, impulse control disorders and (atypical) depression (Viggiano et al., 2004; Fan et al., 2012). Possibly, then, in the face of ongoing stress a lack of IFN-γ and the consequent potentiation of noradrenergic signaling could function as a double-edged sword – at once serving to facilitate the memory process (Tully and Bolshakov, 2010) and predisposing to hyperactivity, with the latter perhaps best viewed as a harbinger of impending allostatic overload. All the same, it is important to recognize that in the present study the motor-modulatory effect of stress was clearly task-specific. Indeed, whereas by the end of the 6-week experiment locomotion in the home-cage was increased among the stressor-exposed mice (predominantly in the KOs), Y-maze testing only 2 days prior revealed no such effect of stress (i.e., on total arm entries). Moreover, it will be recalled that, in the chronically stressed KOs only, Y-maze performance actually tended to correlate *negatively* with total arm entries (retention phase) – a phenomenon that was revealed elsewhere to be associated with good spatial memory performance under chronic stressor conditions (Conrad et al., 2003). Thus, if it can be allowed that enhanced NE utilization did, in fact, contribute to both the cognitive and home-cage motor changes observed in the stressed KOs, then these animals were presumably yet able to harness said noradrenergic drive to their mnemonic advantage and when the peculiarity of the circumstances demanded it.

In addition to monoaminergic imbalances, depression and other stressor-related behavioral disorders may involve disturbances of neuroplasticity, including changes in brain structure and neurotrophin systems (Calabrese et al., 2009; Schmidt and Duman, 2010; Hayley and Litteljohn, 2013). In particular, circulating levels of BDNF were found to be diminished in depressed subjects and to correlate with clinical recovery after treatment initiation (Shimizu et al., 2003; Huang et al., 2008). Similarly, animal studies revealed that chronic stress often diminishes central BDNF levels (Murakami et al., 2005; Luo et al., 2013), whereas BDNF-augmenting strategies (e.g., direct infusions, stimulators) typically induce antidepressant-like behavioral consequences (Shirayama et al., 2002; Schmidt and Duman, 2010; Ye et al., 2011). We were therefore somewhat surprised to find that hippocampal BDNF remained unchanged in the present study upon exposure to chronic stress. Interestingly, though in the minority, a number of studies have reported a lack of or even inverse relationship between hippocampal BDNF and chronic stress (Lucca et al., 2008; Larsen et al., 2010; Hanson et al., 2011). And while not contesting the crucial role of BDNF in memory function, it’s worth noting that hippocampal BDNF has not always been found to positively correlate with memory performance (Muñoz et al., 2010; Bechara and Kelly, 2013). Conceivably, then, germline loss of IFN-γ may have altered the hippocampal neuroinflammatory milieu in such a way that, in the face of chronic stress, upregulated (compensatory) expression of alternative growth factors (e.g., GDNF, NGF, NT-3, IGF-1) and possibly even anti-inflammatory and/or anti-apoptotic messengers (e.g., IL-10 and bcl-2, respectively) could have positively affected memory-related processes independently of BDNF (Bian et al., 2012; Xuan et al., 2014).

Alternatively, since chronic stress has been shown to time-dependently regulate brain regional BDNF expression (Fanous et al., 2010; Xiao et al., 2011; Lakshminarasimhan and Chattarji, 2012; Capoccia et al., 2013), it is possible that our null BDNF findings might be related to the timing of mouse sacrifice relative to stressor initiation and/or termination. Considering that in the present study animals were exposed to a rather lengthy 6-week stressor, a plausible scenario would have the stressor transiently suppressing BDNF levels, only for them to return to baseline by the time of sampling. This contention can be seen to derive at least some support from the home-cage activity data: whereas locomotor activity was significantly elevated after 6 weeks of exposure to stress (and predominantly in the KOs), at the mid-way point of the experiment the opposite was, in fact, noted. In effect, chronic stress time-dependently influenced home-cage motor activity and IFN-γ appeared to modulate the magnitude if not direction of such effects. Without time series data for the neurochemical endpoints we cannot exclude that hippocampal BDNF expression similarly changed across time; this is a definite weakness of the present study and a suggested worthwhile avenue of future research. In addition, we cannot speak to any potential effect of chronic stress or IFN-γ deficiency on BDNF expression at the mRNA level, nor can we rule out the possibility that a more sensitive assay such as ELISA could have detected presumably very subtle changes in BDNF protein expression. However, that Campos et al. (2014) failed to demonstrate an effect of IFN-γ deletion on ELISA-determined BDNF protein levels within the hippocampus and PFC would seem to lend credence to the present null findings, at least in regards to basal state BDNF expression. And finally, our use of genetically engineered mice on a C57BL/6J background might have been an issue, as this mouse strain is considered to be somewhat less stressor-sensitive than certain other strains (e.g., BALB/c; Tannenbaum and Anisman, 2003; Pothion et al., 2004). In this regard, Bergström et al. (2008) showed that chronic mild stress differentially modulates hippocampal BDNF expression in stressor-resilient and stressor-sensitive rats.

## CONCLUSION

To summarize, our data suggest that IFN-γ differentially modulates memory-related processes under normal and chronic stressor conditions. Specifically, in the basal state IFN-γ appears to facilitate hippocampus-dependent spatial memory, probably at least partially due to the cytokine’s involvement in the homeostatic regulation of relevant brain monoamine systems. Under conditions of chronic stress, however, IFN-γ appears instead to restrict potentially adaptive brain noradrenergic and spatial memory responses. While our data do not support a role of hippocampal BDNF in this regard, they do not preclude the possible involvement of other structural and/or functional neuroplastic processes (e.g., IFN-γ-dependent alterations of hippocampal neurogenesis: Baron et al., 2008; Li et al., 2010; Campos et al., 2014); we advocate here for further investigation of this intriguing possibility. More generally, the current findings are aligned with a growing body of work indicating that stressor context can greatly influence the behavioral, immune and neurochemical effects of other cytokine and immune challenges (e.g., IFN-α, LPS, poly I:C; Anisman et al., 2007; Gandhi et al., 2007; Gibb et al., 2008). It should be noted, however, that compensatory neuronal or immunological changes stemming from a lack of IFN-γ during key developmental stages could have contributed to the present findings.

## AUTHOR CONTRIBUTIONS

Conceived and designed the experiments: Darcy Litteljohn and Shawn Hayley. Performed the experiments: Darcy Litteljohn and Eric Nelson. Analyzed the data and wrote the paper: Darcy Litteljohn. Edited and approved the manuscript: Darcy Litteljohn and Shawn Hayley.

## Conflict of Interest Statement

The authors declare that the research was conducted in the absence of any commercial or financial relationships that could be construed as a potential conflict of interest.
